# Effects of nutraceutical diet integration, with coenzyme Q_10_ (Q-Ter multicomposite) and creatine, on dyspnea, exercise tolerance, and quality of life in COPD patients with chronic respiratory failure

**DOI:** 10.1186/2049-6958-8-40

**Published:** 2013-06-21

**Authors:** Stefano Marinari, Maria Rosaria Manigrasso, Fernando De Benedetto

**Affiliations:** 1 Pneumology Department, SS Annunziata Hospital, Chieti, Italy

**Keywords:** Chronic respiratory failure, Coenzyme Q_10_, COPD, Creatine, Dyspnea, Exercise tolerance, Q-Ter, Quality of life

## Abstract

**Background:**

The protein-calorie malnutrition, resulting in muscle mass loss, frequently occurs in severe COPD patients with chronic respiratory failure (CRF), causing dyspnea, reduced exercise tolerance and impaired quality of life.

The cause of this occurrence is an intake-output energy imbalance. A documented deficit of phosphocreatine and reduced mithocondrial energy production can contribute to this imbalance.

Aim of this study is to verify whether a dietary supplementation with creatine and coenzyme Q10, important mitochondrial function factors, is able to influence this mechanism leading to a dyspnea reduction and improving exercise tolerance and quality of life.

**Methods:**

55 COPD patients with chronic respiratory failure (in long term O_2_ therapy), in stable phase of the disease and without severe comorbidities were assigned (double-blind, randomized) to: group A (30 patients) with daily dietary supplementation with Creatine 340 mg + 320 mg Coenzyme Q-Ter (Eufortyn®, Scharper Therapeutics Srl) for 2 months whereas Group B (25 patients) received placebo.

All patients continued the same diet, rehabilitation and therapy during the study. At recruitment (T0) and after 2 months (T1), patients were submitted to medical history, anthropometry (BMI), bioelectrical impedance, arterial blood gas analysis, evaluation of dyspnea (VAS, Borg, BDI, MRC) and functional independence (ADL), 6-minute walk test (6MWT) and quality of life questionnaire (SGRQ). At 6 months and 1 year, a telephone follow up was conducted on exacerbations number.

**Results:**

No significant difference was detected at baseline (T0) in the 2 groups. After 2 months of therapy (T1) the FFMI increased in the daily dietary supplementation group (+ 3.7 %) and decreased in the placebo group (- 0.6 %), resulting in a statistically significant (p < 0.001) treatment difference. Statistically significant treatment differences, favouring daily dietary supplementation group, were also seen for the 6MWT comparison. Group A patients also showed significant: 1) improvement in the degree of dyspnea (VAS: p < 0.05; Borg: p < 0.05; MRC: p < 0.001; BDI1: p < 0.05; BDI3: p < 0.03), and independence level in activities of daily living (p < 0.03); 2) improvement in quality of life in activity section (- 6.63 pt) and in total score (- 5.43 pt); 3) exacerbation number decrease (p < 0.02). No significant differences were found (end of study vs baseline) in group B.

**Conclusions:**

The nutraceutical diet integration with Q-Ter and creatine, in COPD patients with CRF in O_2_TLT induced an increasing lean body mass and exercise tolerance, reducing dyspnea, quality of life and exacerbations. These results provide a first demonstration that acting on protein synthesis and muscular efficiency can significantly modify the systemic consequences of the disease.

## Background

Several studies demonstrated the prognostic importance of body weight and protein-calorie malnutrition in Chronic Obstructive Pulmonary Disease (COPD) patients [1-4], so that malnutrition is an important component of “systemic” problems of international guidelines for the management and the treatment of COPD [5,6].

Over 25 years ago the first observations of this phenomenon showed the high prevalence of underweight status among patients with severe disease, while later studies evidenced the importance of assessing lean body mass reduction often in presence of normal and even increased body weight [7,8].

The principal metabolic causes of malnutrition are: inadequate caloric intake, increased energy expenditure and alteration of protein turnover [9-20].

Moreover the presence of hypoxemia in patients with COPD could determine muscular inefficiency through: reduced muscles aerobic capacity (greater use of anaerobic energy sources, aerobic muscular enzymes reduction, earlier metabolic acidosis) and reduced peripheral O_2_ use (alteration of red blood transport cells/mitochondria, altered O_2_ distribution between operating muscle fibers, cellular oxidative processes inertia) [21].

The effects of these mechanisms on COPD patients’ muscles are on the basis of exercise tolerance reduction, dyspnea increase and poor quality of life [22-27]. Interesting confirmations come from muscular biopsies that showed alterations in number and mitochondrial function [28-32]. Furthermore, studies on muscle metabolism, trough P31-NMR on peripheral muscle, showed an increase in enzymes activity involved in anaerobic glycolysis and reduction of enzymes involved in the oxidative chain. Low concentrations of high energy phosphates (phosphocreatine, ATP-AMP) at rest and slow deposits recovery of phosphocreatine after exercise have been reported [33,34]. Other studies, conducted in chronic diseases such as COPD, demonstrated a deficit of elements such as **c**oenzyme Q_10_ or ubiquinone, which is an essential element for the mitochondrion function that promotes the production of ATP and energy. It is normally synthesized endogenously, but it is frequently reduced in the elderly and in chronic diseases [35].

Based on these assumptions, dietary supplementation studies have been performed with creatine and coenzyme Q_10_ in patients with COPD or chronic heart failure. In particular, creatine and coenzyme Q_10_ seem to have a key role in improving protein turnover and mitochondrial energy production [36-40].

Aim of this study is to demonstrate that, in a group of COPD patients with chronic respiratory failure in O_2_ home therapy, a dietary supplement (CoQ_10_ + creatine) is able to reduce dyspnea and improve exercise tolerance and quality of life.

### Subjects and methods

#### Subjects

We enrolled clinically stable COPD patients with chronic respiratory failure, in O_2_ therapy for at least 3 years and with an optimized pharmacological therapy.

The patients met the following inclusion criteria: patients aged ≥ 60 ≤ 85 years, without exacerbations and hospitalizations (for any reason) in the last month, who could give informed consent were included. During the study, subjects maintained a constant diet and drug therapy (including oxygen).

Exclusion criteria were as follows: smoking habits, home mechanical ventilation, presence of comorbidities such as diabetes, severe heart failure (NYHA 3 and 4), severe renal or hepatic impairment, active or recent neoplastic disease (no cancer treatment for at least 3 years), chronic infections (TB, HIV, serious structural disease of the lungs), chronic systemic steroid therapy, other diseases that could prevent execution of tests according to the study protocol (inability to walk , inability to perform functional testing, etc…).

## Methods

The study protocol described above was approved by the Ethics Committee of "G. D'Annunzio" University of Chieti and Pescara and ASL2 Lanciano, Vasto, Chieti.

In patients who met the inclusion criteria and who provided their written informed consent, at baseline (T0) were done: 1) medical history questionnaire on COPD, duration of O_2_ therapy , number of exacerbations, defined as a rapid change in clinical symptoms of such intensity to require the use of antibiotics and/or systemic steroids, number of hospitalizations in the last year and presence of comorbidities; 2) calculation of Body Mass Index (BMI); 3) complete lung function study: static and dynamic lung volumes, lung diffusion (DL_CO_) (Sensormedics, VMAX); 4) arterial blood gas analysis (ABL 700 series – Radiometer Copenaghen); 5) bioimpendence analysis of body composition (BIA 101, AKERN ) for the evaluation of Fat Free Mass Index (FFMI: FFM/height^2^) [41]; 6) assessment of the degree of resting dyspnea (Medical Research Council - MRC scale and Baseline Dyspnea Index - BDI scale); 7) evaluation of exertional dyspnea (Borg scale, VAS) and measurement of activities of daily living (questionnaire: activities daily living - ADL) [42]; 8) evaluation of exercise capacity (6MWT), according to ATS document, March 2002) in O_2_ therapy [43]; 9) evaluation of the quality of life using Saint George's Respiratory Questionnaire (SGRQ) [44].

Thirty patients received, in double-blind randomized, daily dietary oral supplementation with Creatine 340 mg + 320 mg Coenzyme Q-Ter (Eufortyn®, Scharper Therapeutics Srl) for 2 months (Group A), whereas 25 patients received placebo (Group B).

At time T1 (2 months after the start of therapy) the following parameters were re-evaluated: arterial blood gases analysis, bioimpedence analysis, assessment of the degree of dyspnea at rest and after exercise (VAS, Borg, BDI, MRC) and functional independence (Activities of Daily Living - ADL), 6MWT , and quality of life questionnaire (SGRQ) [44].

After 10 months from the end of therapy, all patients were subjected to a follow up telephone questionnaire on the number of exacerbations in the previous year.

### Study design

It was a randomized, double-blind versus placebo study. The treatments were: Creatine 170 mg + 160 mg Coenzyme Q-Ter (EufortynLios), 2 times a day for 2 months (Group A) or placebo bags apparently identical (Group B).

### Statistical analysis

Statistical analysis was performed by using the SAS (version 9.2) statistical package. The sample size estimate was based on the results of a previous pilot study, assuming a difference of 2.0 % (standard deviation 2.5 %) between groups (daily dietary supplementation versus placebo) for the primary efficacy variable (Free Fat Mass Index) when analyzed with the analysis of covariance (ANCOVA). With normally distributed data and a two-sided α = 0.05, 30 subjects per group were needed to achieve a statistical power of 85% for the primary outcome measure. The safety sample was defined as all subjects who provided consent, were randomized, and it had at least one dose of daily dietary supplementation or placebo. The primary efficacy analysis was performed on the full analysis sample: all subjects in the safety sample who had at least one post-baseline efficacy measurement. Descriptive statistics, mean and standard deviation (SD), were reported for baseline (T0) demographic characteristics and efficacy outcome measures. The primary outcome measure (FFMI) mean was compared between treatment groups using ANCOVA, with the baseline value as a covariate and treatment as a factor. Significance was estimated using the *F*-test at the 5% level. ANCOVA was also performed as an exploratory analysis for the 6MWT comparison between treatment groups. The statistical significance of changes in all efficacy outcomes from baseline to the end of the double-blind phase was assessed using the Paired Sample *t*-Test (exploratory analysis). *p* < 0.05 was considered significant.

## Results

The baseline comparison (T0) between the patient’s characteristics of the two groups didn’t show any significant difference concerning: age, body mass index (BMI), Free-fat mass index (FFMI), arterial blood gas levels in ambient air (partial pressure of O_2_, PaO_2_, partial pressure of CO_2_, PaCO_2_), respiratory function parameters such as forced vital capacity (FVC), forced expiratory volume in 1 second (FEV_1_), residual volume (RV), lung diffusion for carbon monoxide (DL_CO_), degree of dyspnea measured by Medical Research Council (MRC) scale and 6 min walking distance (6MWD) (Table 1).

**Table 1:** Subject characteristics at baseline (safety sample)

	**Group A**	**Group B**	***p***
	**(N = 30)**	**(N = 25)**	
	**Mean ± SD**	**Mean ± SD**	
AGE (years)	73.2 ± 8.7	73.9 ± 7.7	NS
BMI (Kg/m^2^)	30.8 ± 8.8	29.3 ± 7.9	NS
PaO_2_ (mmHg)	58.2 ± 4.9	58.2 ± 6.4	NS
PaCO_2_ (mmHg)	45.0 ± 4.4	43.9 ± 5.4	NS
FEV_1_ (% pred.)	41.7 ± 14.6	44.0 ± 14.6	NS
VC (% pred.)	70.0 ± 22.9	71.3 ± 25.5	NS
RV (% pred.)	116.0 ± 48.7	115.8 ± 51.5	NS
DL_CO_ (% pred.)	58.1 ± 23.6	61.7 ± 33.5	NS
MRC	4.96 ± 0.73	4.04 ± 0.76	NS
6MWT (meters)	236.4 ± 91.4	249.3 ± 74.2	NS

*NS* Not Significant; Independent-Samples *t*-Test.

Patients characteristics showed COPD patients with chronic respiratory failure, advanced age, overweight with fat-free mass preserved, moderate impairment of lung diffusion, high degree of dyspnea and decreased exercise tolerance.

### Body composition and exercise tolerance

From baseline to the end of the double-blind period (T1, two months of therapy), the FFMI (Kg/m^2^) increased in the daily dietary supplementation group (+ 3.7) and decreased in the placebo group (- 0.6 ), resulting in a statistically significant (ANCOVA model *p* < 0.001) treatment difference. Statistically significant treatment differences, favouring daily dietary supplementation group, were also seen for the 6MWT (mtr) comparison (Table 2).

**Table 2:** **Comparison of the free fat mass index (FFMI) and 6MWT distance within-group (Paired sample *t*-Test, T0 vs T1) and between-groups (ANCOVA with baseline value as covariate, Group A vs Group B)**

	**FFMI (Kg/m**^**2**^**)**			**6MWT (mtr)**		
	**T0**	**T1**	**Within-group**	**T0**	**T1**	**Within-group**
	**Mean ± SD**	**Mean ± SD**	***p***	**Mean ± SD**	**Mean ± SD**	***p***
**Group A**	17.1 ± 6.5	20.8 ± 7.4	< 0.001	236.4 ± 91.4	303.7 ± 88.1	< 0.001
**Group B**	19.3 ± 5.5	18.7 ± 4.3	0.093	249.3 ± 74.2	203.9 ± 88.1	0.021
**Between**- groups ANCOVA *p*	-	< 0.001	-	-	< 0.001	-

**Figure 1:**
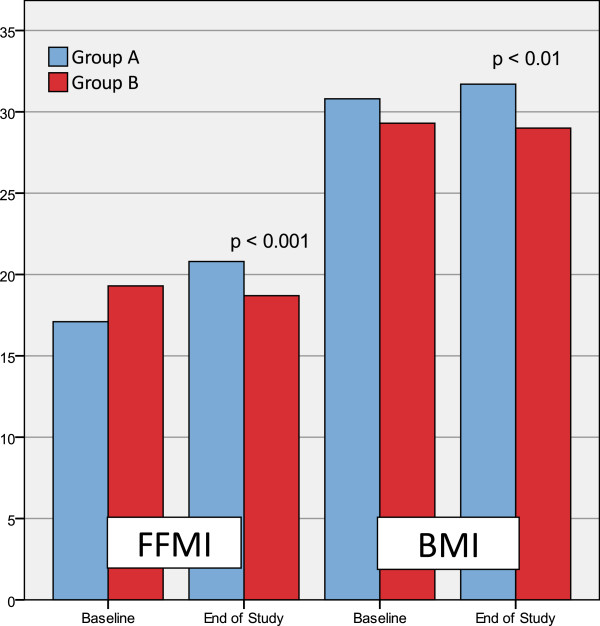
**End of study comparison between the two groups of the Fat Free Mass Index (FFMI) and Body Mass Index (BMI).** After two months of therapy FFMI (*p* < 0.001) and BMI (*p* < 0.01) were higher in patients treated with dietary supplementation (group A).

During the treatment period the FFMI increased, with consequent BMI increase, only in patients treated with dietary supplementation (group A), in comparison to patients treated with placebo (group B). After two months of treatment the FFMI and BMI comparisons between groups showed a statistically significant difference (Figure 1).

### Dyspnea

The evaluation of dyspnea degree using different scales (Borg, VAS, MRC, BDI) showed a significant improvement of dyspnea in Group A at T1 (VAS: 3.81 ± 2.12 vs 2.94 ± 2.34 *p* < 0.05; Borg: 2.96 ± 1.59 vs 2.31 ± 1.67, *p* < 0.05; MRC: 3, 96 ± 0.73 vs 3.0 ± 1.06, *p* < 0.000; BDI1: 1.65 ± 1.11 vs 2.37 ± 0.82, *p* < 0.05; BDI3: 1.82 ± 1. 03 vs 2.48 ± 0.73, *p* < 0.03). BDI2 scale showed no significant differences in group A, while all dyspnea evaluations in group B were not significant.

**Figure 2:**
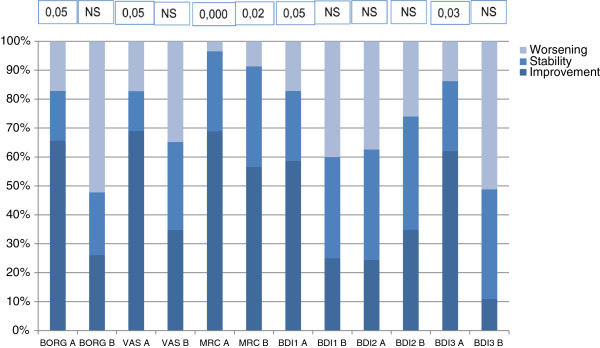
**Comparison (T0 vs T1) between rate of patients of two groups with improvement, stabilization and worsening dyspnea (Borg, VAS, MRC and BDI scale), before and after treatment.** Significant higher rate of stable or improved patients in group A.

A higher rate of group A patients showed improved or stable dyspnea values (26.1% vs 65.6% BORG; VAS: 68,9% vs 34.8%; MRC: 69% vs 56.5%; BDI1: 58.7% vs. 21.7%; BDI3: 62.1% vs. 11.1%) (Figure 2).

### Functional independence degree

**Figure 3:**
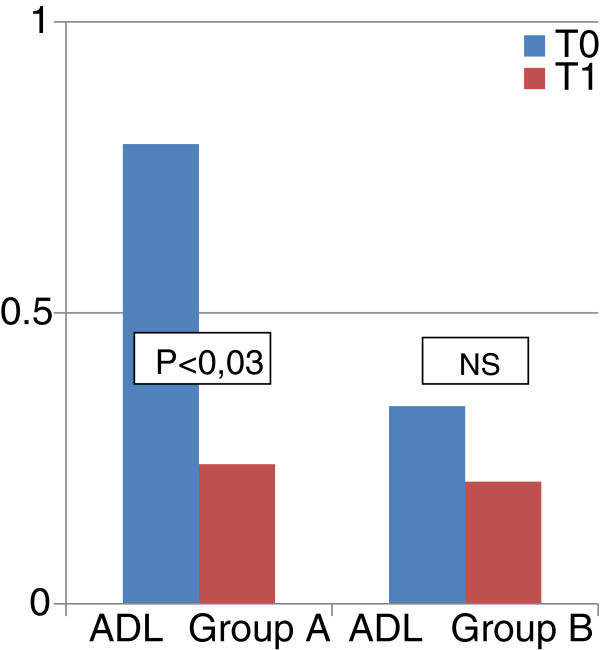
**Comparison (T0 vs T1) between Functional Independence Level (Activities of Daily Living- ADL) of two groups.** Significant improvement in the independence level in activities of daily living in group A at T1. No significant difference was found in group B.

The evaluation of performance index with Activities of Daily Living scale (ADL) showed a significant improvement in the independence level in activities of daily living in group A at T1 (0.79 ± 1.23 vs. 0.24 ± 0.51, *p* < 0.03). No significant difference was found in group B (Figure 3).

### Quality of life

**Figure 4:**
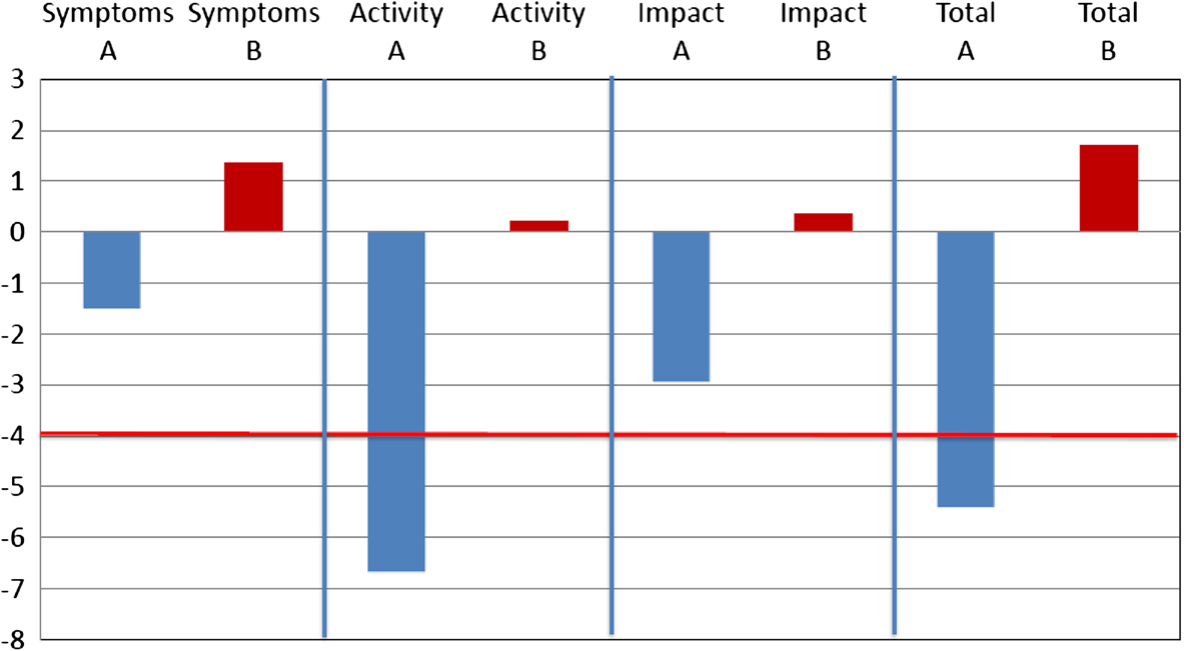
**Comparison (T0 vs T1) between Quality of Life questionnaire (SGRQ) in two groups.** A significant improvement in quality of life (cut-off improvement > 4 points) in group A in activities section (- 6.63 pt) and total score (- 5.43 points). No significant change in group B.

Quality of life evaluation with the SGRQ at T0 and T1 showed a significant improvement in quality of life (cut-off improvement > 4 points) [43] in group A in activities section (- 6, 63 pt) and total score (- 5.43 points). No significant change in group B (Figure 4).

### Exacerbations

**Figure 5:**
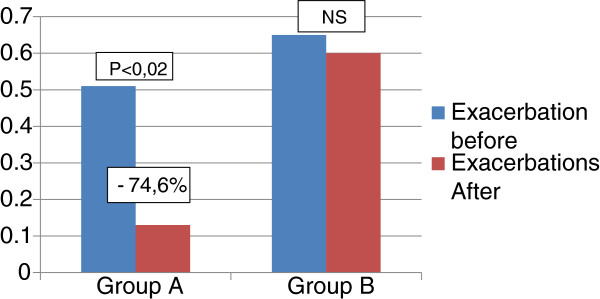
**Comparison between exacerbations rate before (1 year before treatment) and during the study (1 year following the start of treatment) in the two groups.** Significant reduction of exacerbations in group A.

The exacerbations rate in the year following the start of therapy was significantly decreased in group A (0.51 ± 0.78 vs 0.13 ± 0.35, *p* < 0.02), with a reduction of 74.6%, compared to baseline. In the placebo group (Group B) no statistically significant difference (0.65 ± 1.11 vs 0.60 ± 0.72, NS) was demonstrated (Figure 5).

## Discussion

The results of this study demonstrate that in severe stable COPD patients with chronic respiratory failure, independently of body mass alterations (evident body weight and/or lean body mass reduction), dietary supplementation with a Creatine-Q-Ter compound determines a variation of body mass with lean body mass increase. This change is associated with improvement in exercise capacity and dyspnea, and reduction of exacerbations rate. In these patients such effect leads to a functional independence in the activities of daily living and quality of life increase.

### Malnutrition and COPD

It has been widely demonstrated [9-20] that the protein-calorie malnutrition and the underweight status are a constant manifestation of some patients with severe COPD, and it is clear that their cause is the imbalance among inadequate caloric intake, energy expenditure increase and protein turnover alteration.

The determinants of imbalance are numerous. On the one hand dyspnea influences nutrition through an after-meal diaphragm movement restriction, as well as altered appetite sensation, caused by leptin levels reduction, that is related to systemic inflammation [45] and can contribute to a reduced caloric intake. On the other hand, the energy expenditure increase is supported by metabolism increase at rest and after exercise, alteration of the sympathetic regulation and drugs influence. Moreover, systemic inflammation, as demonstrated by the increase in cytokines , such as TNF-alpha, plays a key role in protein turnover. In this regard, an increased apoptosis, that contributed to the exercise limitation, not explained by other causes such as hypoxemia, tobacco smoking and steroid therapy, was detected in the muscle cells of malnourished COPD patients [46].

Finally, oxidative stress, caused in particular by cigarette smoke and neutrophils during exacerbations, is together with chronic inflammation, a principal pathogenetic mechanism for COPD development.

### Muscle and mitochondrial alteration

Other mechanisms, which are closely related to protein-calorie malnutrition, concerning structural and biochemical alterations of the peripheral muscles, have been demonstrated in COPD patients who also showed muscular fibers and mitochondria alterations.

There have been several experimental demonstrations of decreased muscle mass in patients with COPD, even in the relatively early stages of the disease, which was reflected in a subsequent reduction of respiratory and peripheral muscle strength [47].

Moreover, studies on muscle biopsies clarify that in the muscles of these patients there is a qualitative adaptation mechanism, like a greater presence of type II muscle fibers, able to easily use an anaerobic metabolism, compared to type I fibers with aerobic metabolism [48]. This change reflects a reduced aerobic capacity of the muscle of COPD patients, especially if hypoxemic, with a greater use of renewable anaerobic energy and reduced peripheral oxygen consumption [21,49].

Interesting results were achieved by investigations about muscular changes related to muscle mitochondrial energy production.

Muscle mitochondrial alterations have been demonstrated both in terms of reduced density [31] and in function of cytochrome C [28].

Studies performed in underweight and normal weight COPD patients (muscle biopsies) showed a reduction of mitochondrial function in underweight patients caused by abnormal electron transport and consequent worsening of muscular endurance [50]. Puente-Maestu et al. confirmed this phenomenon in COPD muscular biopsies [29], also at the early stage of disease, with an altered mithocondrial electron transport and an increase in ROS, that is a possible cause of systemic oxidative muscular damage. On the other hand, the studies of Ribera [51], Hamaoka [52], Layec [53] and Picard [54] come to a different conclusion: in fact, while confirming the reduced muscular efficiency, they did not show alterations of mitochondrial function and energy production.

### Effects of changes in dietary supplementation

Attempts to significantly influence body composition through a diet variation, with increase both in carbohydrates [55] and in lipids [56], have obtained controversial results, also in COPD patients undergoing rehabilitation treatment [57].

In particular, in a study by Steinert et al., carbohydrate supplementation has been shown to improve exercise capacity in underweight COPD patients compared to control, despite a body weight increase, while normal-weight COPD patients achieved an improved exercise capacity related to the supplement of carbohydrates [58]. Cai et al. [56], inversely, in about 60 malnourished COPD patients showed that respiratory function (FEV_1_ and ventilation/min), blood gases and respiratory quotient significantly improved after a 3 week of high lipid- low carbohydrate supplementation diet , compared with a traditional diet (high carbohydrate).

Recently, the Engelen’s studies [59,60] achieved some interesting conclusions, demonstrating an increased anabolic response to milk proteins, in particular casein (oral and intravenous) associated with reduced splanchnic extraction of essential amino acids in normal-weight COPD patients during and after exercise.

### Effects of a dietary supplement

Interesting results have been observed with the aminoacids diet supplementation [61,62]. Essential aminoacids in fact promote and enhance protein synthesis and increase muscular tissue growth [63].

Sarcopenic COPD patients who underwent rehabilitation program and 12 weeks essential aminoacid supplementation, increased muscle mass, muscle strength, physical activity, cognitive status and quality of life [61,62] and improved muscle metabolism with a lower lactate concentration.

Other clinical trials, conducted in healthy subjects showed that creatine diet supplementation can increase lean body mass and physical performance through increased deposition of phosphocreatine (phosphorylated form of creatine) in muscle and brain, that reduces the loss of ATP, stimulates protein synthesis, reduces protein catabolism, and stabilizes cell membranes [37]. Studies in patients with chronic heart failure (CHF), in whom phosphocreatine / ATP ratio is a stronger prognostic factor than the degree of impairment [64], showed that dietary supplementation with creatine increases creatine phosphate resynthesis and is associated with increased muscular mass strength and power [65,66].

On this evidence Fuld’s study [40] showed that, in 38 severe COPD patients, selected to receive creatine monohydrate or placebo during pulmonary rehabilitation, creatine diet supplementation increased lean body mass, peripheral muscular strength, endurance and health status.

Recently several investigators focused on the study of a very important molecule in the muscular energy production: Coenzyme Q_10_ or ubiquinone. It is a powerful antioxidant, synthesized by human cells (mainly concentrated in the myocardium, liver and adrenal gland), which has a key role in the electron transport chain and consequently in the energy synthesis (ATP) at mitochondrial level. It stabilizes also cell membranes, where it acts as a scavenger of ROS. One of the principal limitations of CoQ_10_ clinical use is its reduced oral absorption, currently passed by CoQ_10_-terclatrat to Q-Ter (multicompoundCoenzima Q_10_ based, obtained through mechano-physic CoQ_10_ activation with carrier and bioactivator function materials) 200 times more water-soluble and therefore bioavailable.

Studies conducted in chronic heart failure patients showed its important role in improving cardiac output and oxygen consumption [67,68] and also as an independent predictor of mortality [69] actually investigated in a multicenter large International study (Q-symbio) [70].

Even COPD patients showed CoQ_10_ reduction [35] that may contribute to the reduction of muscular efficiency observed in these patients.

An interesting clinical trial in patients with chronic heart failure [71] assuming CoQ_10_ and creatine led to the conclusion that such supplementation improves exercise tolerance and quality of life of patients with severe left ventricular cardiac dysfunction.

## Conclusions

This pilot, double-blind, placebo-controlled trial, has been conducted in BPCO patients with chronic respiratory failure and long term oxygen therapy in order to verify the effectiveness of dietary supplementation of creatine and Qter in reducing dyspnea and improving exercise tolerance and quality of life.

These results show that such supplementation, safe and well tolerated, increases lean body mass level and exercise capacity of these patients, reducing dyspnea. These effects, likely determined by the greater availability of elements, crucial for muscular energy production, allow patients to improve their functional independence with more opportunities to perform activities of daily living and to achieve a better quality of life. Disease stabilization, increased patient functional activity, oxidative load decrease , cause a significantly reduction of disease exacerbations, and consequently of respiratory functional decline and mortality, in these patients.

This study, of course, has some limits, consequent to a small sample size, lack of data on the elements plasma concentration, and finally a limited prospective time observation. The results of this study, however, provide a demonstration that, in COPD patients in O_2_TLT, nutraceutical supplement acting on protein synthesis and muscular efficiency, can significantly influence the systemic consequences of the disease.

Further prospective studies with larger population samples are needed to confirm the effectiveness of this nutritional intervention.

## Competing interests

The authors declare that they have no competing interests.
